# Comprehensive analysis of microbiota signature across 32 cancer types

**DOI:** 10.3389/fonc.2023.1127225

**Published:** 2023-03-08

**Authors:** Xia Yang, Huimin An, Yongtao He, Guoxiang Fu, Zhinong Jiang

**Affiliations:** Department of pathology, Sir Run Run Shaw Hospital of Zhejiang University School of Medicine, Hangzhou, China

**Keywords:** microbiota signature, survival outcomes, genomic features, immune profiles, bacteria in cancer, the Cancer Genome Atlas

## Abstract

Microbial communities significantly inhabit the human body. Evidence shows the interaction between the human microbiome and host cells plays a central role in multiple physiological processes and organ microenvironments. However, the majority of related studies focus on gut microbiota or specific tissues/organs, and the component signature of intratumor microbiota across various cancer types remains unclear. Here, we systematically analyzed the correlation between intratumor microbial signature with survival outcomes, genomic features, and immune profiles across 32 cancer types based on the public databases of Bacteria in Cancer (BIC) and The Cancer Genome Atlas (TCGA). Results showed the relative abundance of microbial taxa in tumors compared to normal tissues was observed as particularly noticeable. Survival analysis found that specific candidate microbial taxa were correlated with prognosis across various cancers. Then, a microbial-based scoring system (MS), which was composed of 64 candidate prognostic microbes, was established. Further analyses showed significant differences in survival status, genomic function, and immune profiles among the distinct MS subgroups. Taken together, this study reveals the diversity and complexity of microbiomes in tumors. Classifying cancer into different subtypes based on intratumor microbial signatures might reasonably reflect genomic characteristics, immune features, and survival status.

## Introduction

Various and complex microorganisms inhabit the human body, composing what is called the human microbiota (1, 2). Evidence shows the interaction between the human microbiome and host cells plays a central role in multiple physiological processes and organ microenvironments (3, 4). For instance, the gut microbiota regulates host metabolism and the immune system through putative-specific microbes, metabolites, and toxins (5–7). Certain bacterial components of the human microbiota can drive tumorigenesis and development in various cancers. Numerous evidence found that the bacterium Helicobacter pylori contributes to atrophic gastritis, peptic ulcers, gastric cancers *via* Wnt/b-catenin pathway, and chronic inflammatory response (8–10). Fusobacterium nucleatum has been found highly abundant in colorectal cancer (CRC) tumors and metastasis tissues than in matched normal tissues, which was associated with poorer prognosis for CRC patients (11–13).

Microbial dysbiosis contributes to tumor susceptibility through complex mechanisms, including inducing tumorigenesis and progression through inflammation, remodeling immune and stromal cells in the tumor microenvironment, and interfering with anticancer drug pharmacodynamics (14–17). An analysis of 16 rRNAs found in stool discovered that the structure and function of gut microbiota in patients with lung cancer were unbalanced, and the imbalance between firmicutes and Bacteroides contributed to tumorigenesis and progression of lung cancer (18, 19). Moreover, the diversity of gut microbiome has been found to positively correlate with the efficacy of immunotherapy in various cancer types (20, 21). Fusobacterium nucleatum is an oral anaerobe that has been found to be prevalent in colorectal cancer and breast cancer, which promoted tumor growth and metastatic progression by attaching tumor-displayed Gal-GalNAc *via* Fap2 (13, 22, 23).

Although the majority of related studies focus on gut microbiota (24–26), several studies have recently characterized the existence, metabolic activity, and functional importance of intra-tumoral microbiota in various cancers (23, 27–30). Exploring the alteration in the microbial community derived from human tissues and organs will help us better understand the occurrence, progression, and therapeutic approaches for tumors. Bacteria in Cancer (BIC, http://bic.jhlab.tw/ ) reveals a collection of curated, decontaminated tissue-resident microbiota of 32 cancer types based on samples from the TCGA program (31). The microbial signatures of tumor and normal samples from 32 cancer types can be estimated from BIC at different taxonomic levels, which provides an excellent and powerful resource for studying the abundance and alternation of microbial components in various cancers.

The objective of the current study was to investigate the microbiota profile across 32 cancer types. First, we identified the relative abundance of microbiota in tumor tissues compared to normal tissues at various taxonomic levels. Next, we estimated the prognostic value of various microbial compositions. Then, three microbiome-based clusters were determined by NMF clustering analysis based on candidate prognostic microbiota. A microbial-based scoring system (MS) was established by applying the least absolute shrinkage and selection operator (LASSO) regression algorithm, a machine learning-based approach that selected features that were predictive of survival outcomes. Furthermore, the correlation between MS and patients’ survival outcomes, genomic features, and immune profiles was further investigated by combining the BIC microbial profile with the genomic and clinical data from the TCGA cohort.

## Materials and methods

### Data acquisition from BIC and TCGA

**Figure 1:**
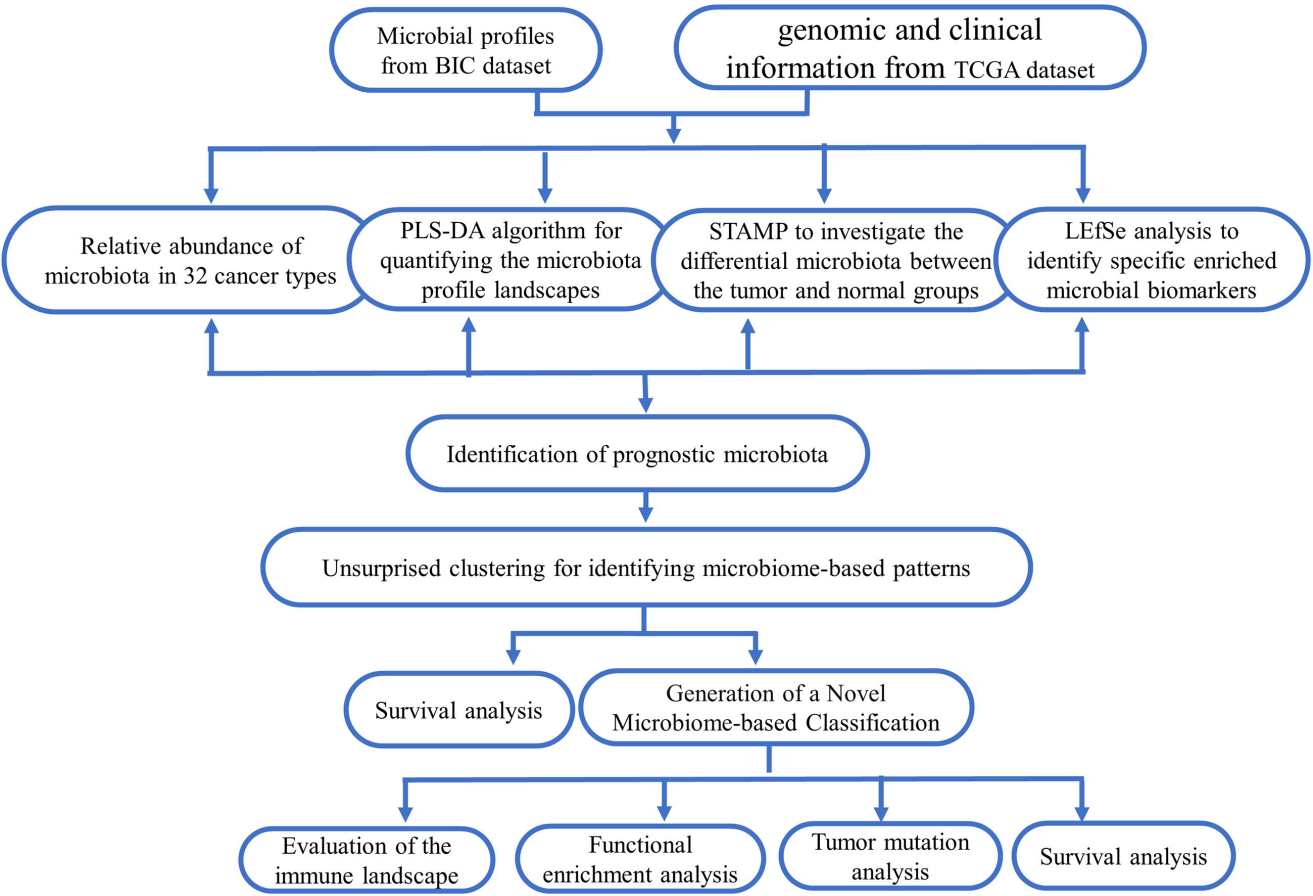
Schematic overview of the study design and workflow.

In this study, the microbiota profiles of samples from 10,362 cases (including 9,687 tumor tissues and 675 adjacent normal tissues across 32 cancer types) at the phylum, class, order, family, and genus levels were obtained from the BIC database (http://bic.jhlab.tw/ ); the corresponding genomic and clinical data for patients were obtained from the TCGA dataset (https://portal.gdc.cancer.gov/repository ). **Figure 1** displays an overview of the study design, and the information on included tumor types is summarized in **Table S1**.

### Microbiota abundance analysis of tumor and normal tissues

First, we calculated the relative abundance of microbiota between the tumor and normal groups at the phylum, class, order, family, and genus taxonomic levels. Then, we applied Partial least squares discrimination analysis (PLS-DA) to visualize the microbiota profile landscapes between tumor and normal samples by using the package “mixOmics”. Next, we performed a statistical analysis of metagenomic profiles (STAMP) to investigate the overall differences in microbiota profiles between the tumor and normal groups. Moreover, Linear discriminant analysis effect size (LEfSe) analysis was used to identify specific enriched microbial biomarkers for each group. Linear discriminant analysis (LDA) was further applied to evaluate the microbial effects for different groups.

### Analysis of global microbiota profiles among 31 cancer types

We first identified the cross-tumor abundant microbiota taxa at the phylum, class, order, family, and genus levels, respectively. Then, we calculated the relative abundance of microbiota across 31 tumor types (except for GBM, which only had normal tissues in the BIC program) at the phylum, class, order, family, and genus taxonomic levels.

### Non-negative matrix factorization clustering analysis

Patients with survival data and follow time ≥ 30 days were chosen for survival analyses. First, we investigated the prognostic significance of microbiota by performing a univariate Cox proportional hazards model. Then, NMF was applied to identify distinct microbiome-based clusters based on the abundance of candidate prognostic microbes. The optimal number of clusters and their stability were determined by the consensus clustering algorithm. The R package “NMF” was used to perform the consensus clustering (32).

### Generation of a novel microbiome-based classification

To quantify the microbiome features of individual patients, we explored a novel microbial-based scoring system (MS) to investigate the microbiota features of the individual patients. Specifically, candidate prognostic microbiomes were chosen from the Lasso regression algorithm to construct the microbial scoring system. The MS was calculated by the corresponding coefficients of selected microbiota signatures:

MS = Σ*i* Coefficient (microbiota)* Abundance (microbiota)

Where *i* represents the selected microbial signatures.

### Gene set variation analysis and gene set enrichment analysis

GSVA enrichment analysis was performed to investigate the variation in biological processes between different MS subgroups by using “GSVA” R packages (33). The gene sets of “c2.all.v2022.1.Hs.symbols” and “h.all.v2022.1.Hs.symbols” were downloaded from the MSigDB database. Adjusted P value <0.05 was considered statistically significant. GSEA was used to explore the signaling enrichment between different MS subgroups by applying the “Clusterprofile” R package (34). The FDR q < 0.25 and P < 0.05 were considered statistically significant.

### Estimation of TME cell infiltration

The ssGSEA algorithm was used to quantify the relative abundance of 29 immune cell types in the TME (35, 36). The relative abundance of each immune cell type in each sample was represented by the enrichment scores that were calculated by the ssGSEA analysis. The CIBERSORT algorithm was applied to analyze the compositions of 22 types of tumor-infiltrating immune cells among different MS subgroups (37).

### Significantly mutated genes and tumor mutation burden in different MS subgroups

Using the R package maftools (38), the overall mutation landscape was summarized in patients with high and low MS subgroups in the TCGA cohort. Then, TMB scores based on the TGCA somatic mutation data were calculated to evaluate the mutation status between different MS subgroups.

### Statistical analysis

Student’s t-tests were applied to analyze normally distributed variables and the Wilcoxon rank-sum test was performed to evaluate non-normally distributed variables. One-way ANOVA and Kruskal-Wallis tests were used to conduct difference comparisons of more than two groups. Kaplan-Meier survival analysis and Cox proportional hazards model were chosen to investigate the prognostic significance of microbiota and microbiota-based subtypes by applying the survival and survminer packages. All statistical P values were two sided, with p < 0.05 being statistically significant. All data processing was done in R 4.0.5 software.

## Results

### Differential microbiota signatures in tumor and normal tissues

**Figure 2:**
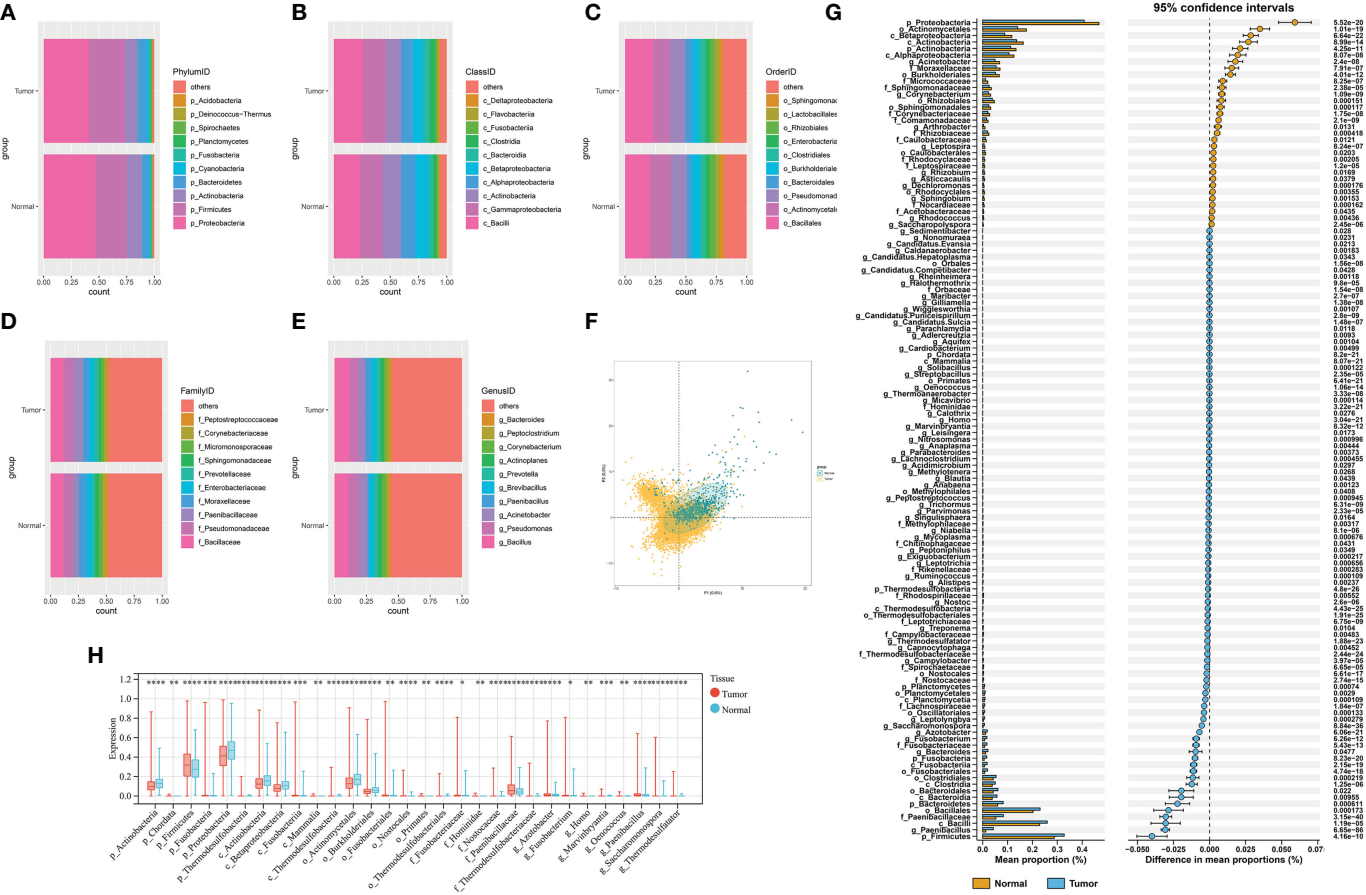
**(A–E)** The differential composition of microbiota in tumor and paired normal tissues at the phylum, class, order, family, and genus levels, respectively. **(F)** PLS-DA plot of microbe signature in tumors and adjacent normal tissues. **(G)** STAMP plot of the differential microbiota composition signatures between tumors and adjacent normal tissues. **(H)** The top 30 differential microbial taxa in tumor and paired normal tissues. * P < 0.05, ** P < 0.01, *** P < 0.001 and **** P < 0.0001.

Overall, we collected and integrated the microbiota profile and clinical characteristics of 10,362 samples from 32 cancer types. A total of 47, 56, 127, 303, and 1,607 microbial taxa were obtained for each sample at the phylum, class, order, family, and genus levels, respectively. First, the average relative abundance of differential microbiota between tumor and normal tissues was calculated (**Figures 2A–E**), which shows the phylum, class, order, family, and genus levels, respectively. Then, the PLS-DA plot exhibited the microbiota profile landscapes between the tumor and normal samples (**Figure 2F**). We next explored the differential microbial compositions for each group. Overall, 8, 10, 17, 25, and 66 differential microbial components were found between tumor and normal tissues at the phylum, class, order, family, and genus levels, respectively (**Figures 2G**).

At the phylum level, Chordata, Firmicutes, Fusobacteria, Bacteroidetes, and Planctomycetes were the main bacteria groups in tumor samples, while Proteobacteria and Actinobacteria were the main groups in normal tissues. At the class level, the microbial composition of Actinobacteria, Alphaproteobacteria, and Betaproteobacteria were significantly abundant in normal tissues, whereas Fusobacteriia, Clostridia, Bacilli, and Bacteroidia were increased in tumor samples. Other differential microbial composition signatures between tumor and normal tissues are shown in **Figure 2G** and **Table S2**. The top 30 differential microbial taxa between tumor and normal tissues are shown in **Figure 2H**.

### LEfSe analysis helps to identify tumor- and normal-enriched microbiota

**Figure 3:**
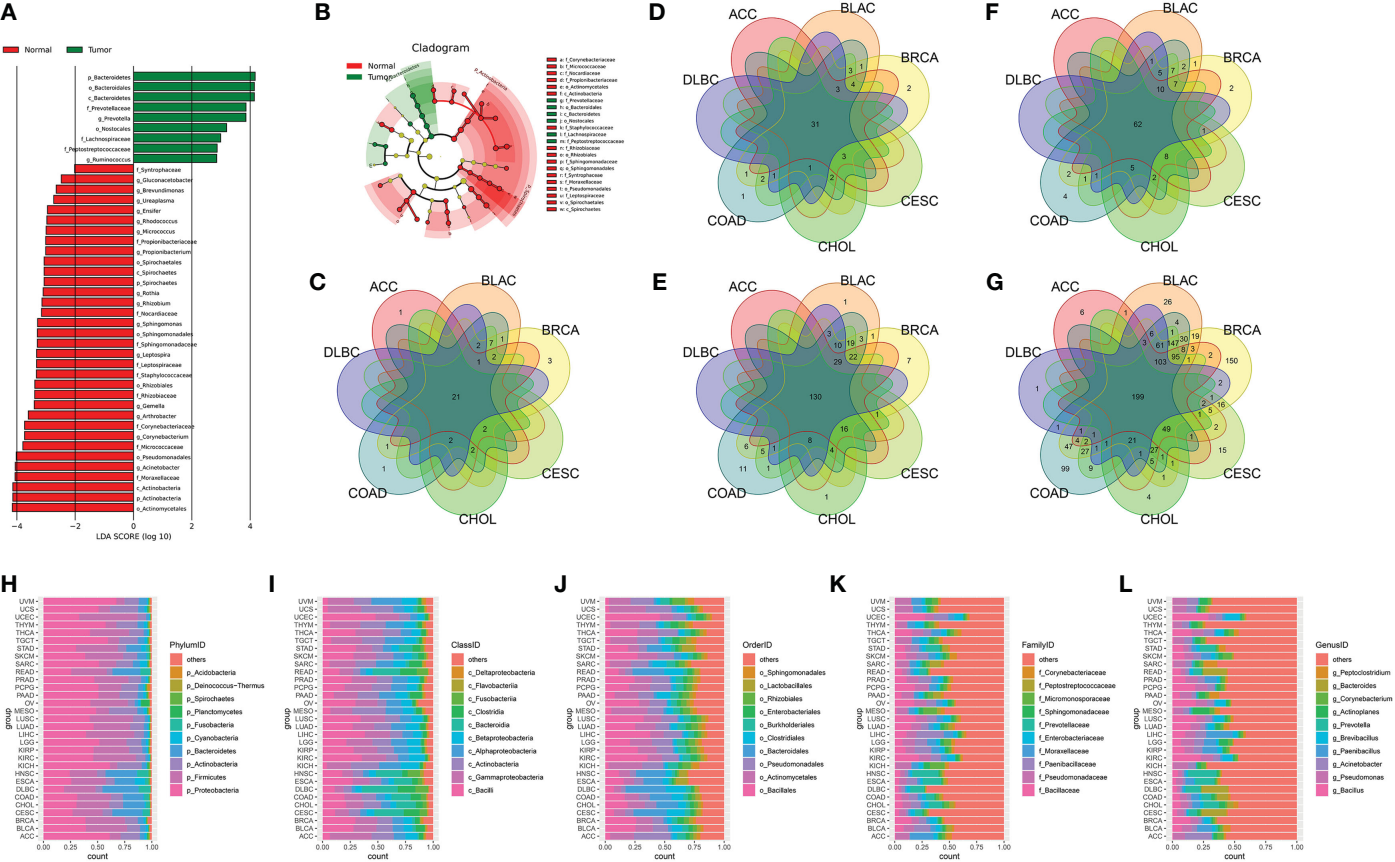
**(A)** The LDA score of specific microbial taxa in the tumor and normal group. **(B)** Cladogram showing tumor- and normal-enriched microbial taxa. **(C–G)** Venn diagram of the cross-tumor abundant microbiota taxa at the phylum, class, order, family, and genus levels, respectively. **(H–L)** The top 10 most differential microbial taxa across 31 cancer types at the phylum, class, order, family, and genus levels, respectively.

The LDA score of specific microbial taxa in the tumor and normal groups showed the compositional abundances of p_Bacteroidetes, c_Bacteroidetes, o_Nostocalles, f_Prevotellaceae, and g_Ruminococcuss were higher in tumor tissues, while the compositional abundances of p_Actinobacteria, c_Actinobacteria, o_Actinomycetales, f_Moraxellaceae, and g_Acinetobacter were enriched in normal tissues (**Figure 3A** and **Table S3**). Cladogram further showed the distinct tumor- and normal-enriched microbiota (**Figure 3B**). Next, we analyzed the cross-tissue abundant microbiome in 32 cancer types at the phylum, class, order, family, and genus levels, respectively. The average relative abundance of microbiota among 31 cancer types (except for GBM, which only had normal tissues in the BIC program) was further explored, which shown in **Figures 3C–G**. Moreover, the top 10 most differential microbial taxa across 31 cancer types were shown in **Figures 3H–L** at the phylum, class, order, family, and genus levels, respectively.

### Exploring prognostic microbiota and different clusters mediated by microbiota

**Figure 4:**
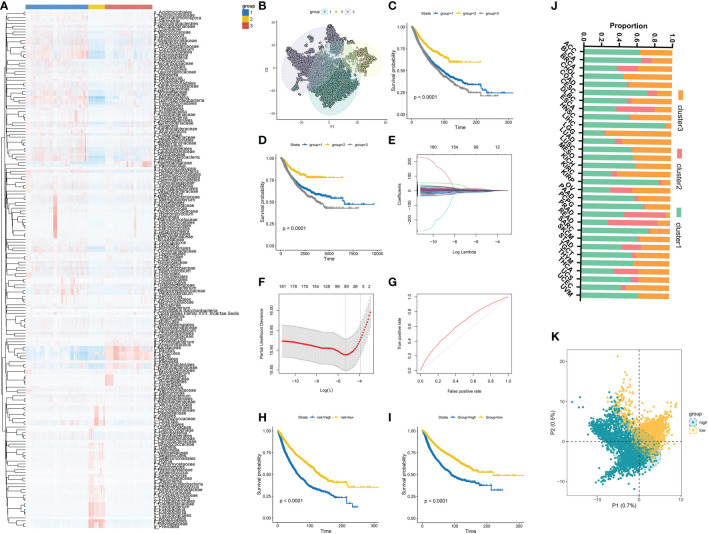
**(A)** Three distinct clusters were established based on the abundance of prognostic microbial taxa. **(B)** tSNE plot showed the performance of microbiome-based clusters to distinguish tumor samples. **(C, D)** Kaplan-Meier plots of OS and DFS among different clusters in the TCGA cohort. **(E, F)** 64 prognostic microbial taxa were further selected by the Lasso regression algorithm. **(G)** Receiver-operating characteristic curves of the microbial signature predicted performance to predict OS. **(H, I)** Survival analyses for OS **(D)** and DFS **(E)** among different MS groups in the TCGA cohort. **(J)** The distribution of different clusters across 31 cancer types in the TCGA cohort. **(K)** PLS-DA plot of microbial signature in high and low-MS groups.

We first investigated specific candidate microbial taxa that correlated with survival outcomes by integrating the microbial abundance profile from BIC and survival information from TCGA. The results showed that a total of 182 microbiota were significantly associated with overall survival (OS) (**Table S4**) and 112 microbiomes were significantly associated with disease-specific survival (DSS) (**Table S7**). Then, Consensus Clustering analysis of the NMF algorithm was applied to classify patients with qualitatively different clusters based on the abundance of 182 prognostic microbiota (**Figure 4A**). Three distinct microbiome-based clusters were eventually identified, including 4,592 cases in cluster 1, 1,223 cases in cluster 2, and 3,460 cases in cluster 3. A tSNE plot further showed the performance of microbiome-based clusters to distinguish tumor samples in the TCGA cohort (**Figure 4B**). A Kaplan-Meier plot revealed a particularly prominent survival advantage in cluster 2, whereas the worst prognosis was found in cluster 3 (**Figures 4C, D**). The proportion of distinct clusters in 31 tumor types are shown in **Figure 4J**.

### Construction of microbial signature and microbiome-based scoring system

**Figure 5:**
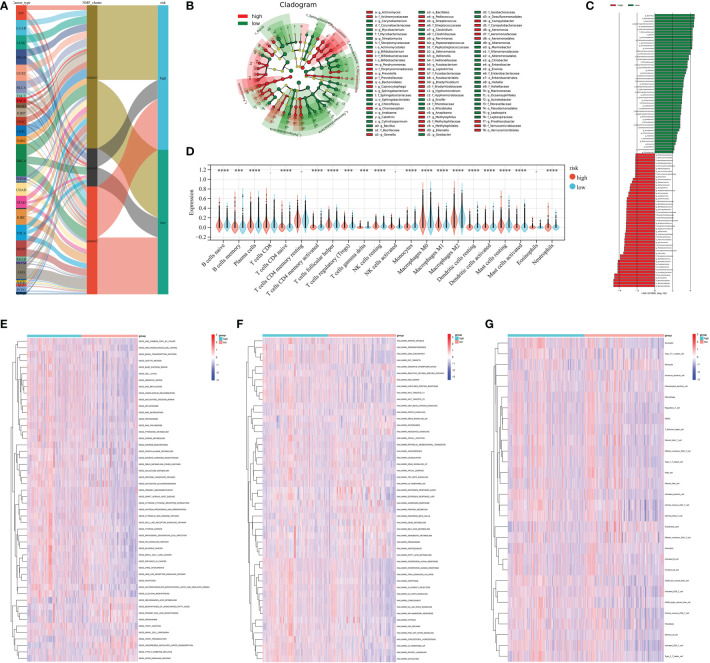
**(A)** Alluvial diagram showing the changes in cancer types, clusters, and MS subtypes. **(B, C)** LEfSe analysis identifying differential microbiota profiles between low- and high-MS groups. **(D)** Cibersort reveals the abundance of each TME infiltrating cell between the low- and high-MS groups. **(E)** Differences in KEGG pathways **(E)** and cancer hallmarks **(F)** between the low- and high FS groups. **(G)** Associations between microbial signature and the ssGSEA scores of tumor microenvironment cell infiltration. *** P < 0.001 and **** P < 0.0001.

The Lasso regression algorithm based on the 182 prognostic microbiota was performed to find candidate microbial signatures (**Figures 4E–G**). A total of 64 selected microbial taxa were identified from the Lasso regression algorithm (**Table S5**). Then, a novel microbiome-based scoring system was constructed to quantify the microbial profiles of individual patients, which we termed as microbial score (MS). Consistent with the NMF clustering analysis, two distinct MS subgroups were found and we named these two subgroups MS-low and -high. Further survival analysis indicated significant prognostic differences between the low- and high-MS subgroups (**Figures 4H, I**). The performance of 64 selected microbial signatures to classify different MS groups was further analyzed by using PLS-LA (**Figure 4K**). To better illustrate the association between the established MS with prognosis, an alluvial diagram was applied to visualize the attribute changes of individual patients (**Figure 5A**). LEfSe analysis was further performed to identify specific enriched microbial biomarkers for high- and low-MS groups (**Figures 5B, C**, and **Table S6**).

### TME cell infiltration characteristics in distinct MS subgroups

**Figure 6:**
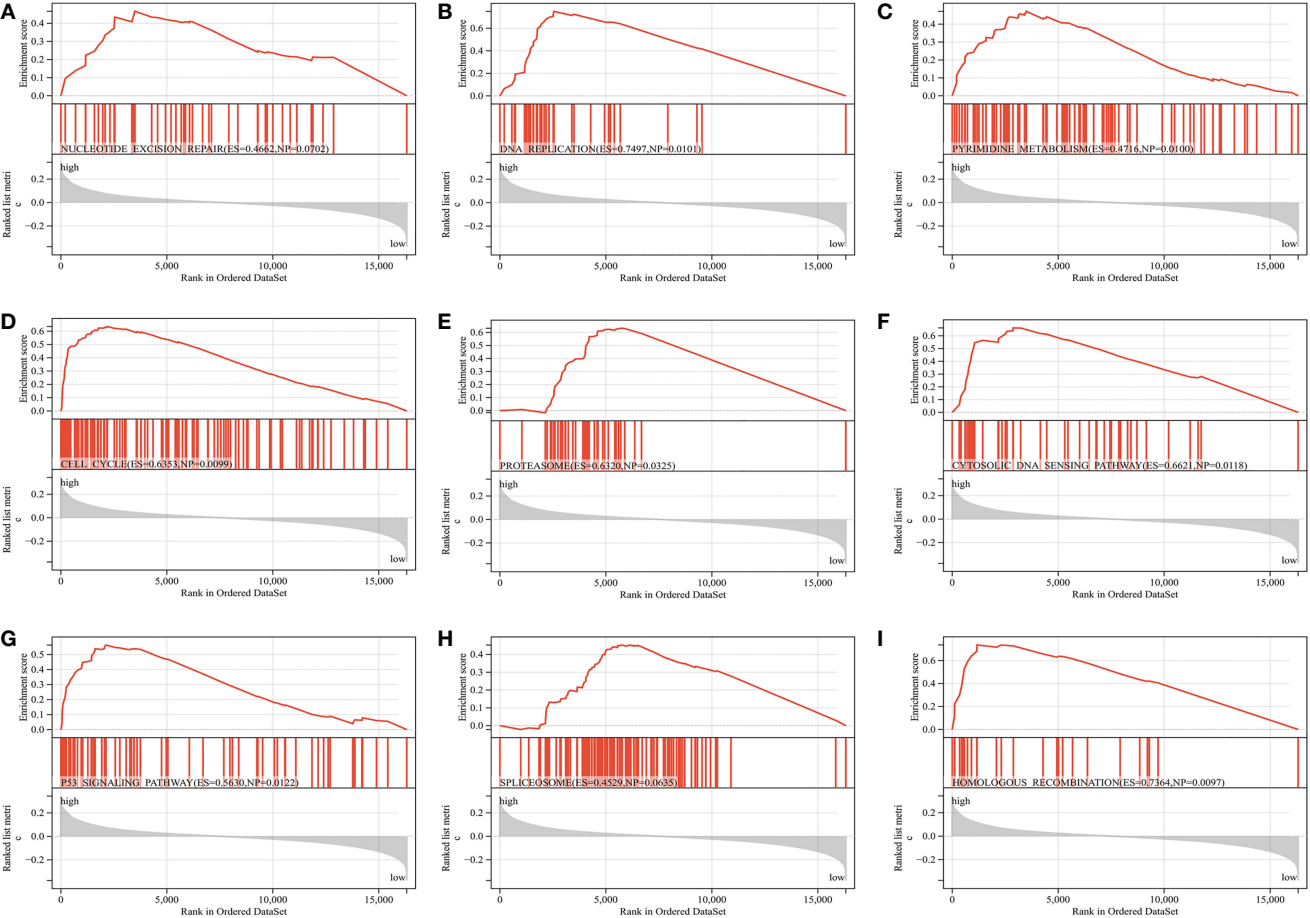
GSEA reveals the key differential signaling pathway between the MS-low and MS-zero subgroups **(A–I)**.

The CIBERSORT algorithm was used to show the differences in the compositions of tumor microenvironment (TME) immune cell types between distinct MS subgroups. As shown in **Figure 5D**, remarkable differences in immune cell infiltration were observed between the high and low-MS groups, which suggested that intratumor microbiota plays an inevitable role in tumor microenvironment immune profiles. Furthermore, the GSVA algorithm showed significant differences in KEGG pathways, cancer hallmarks, and immune profiles among the distinct MS subgroups (**Figures 5E–G**). The GSEA algorithm showed nucleotide excision repair, cell cycle, DNA replication, homologous recombination, cytosolic DNA sensing pathway, pyrimidine metabolism, proteasome, spliceosome, and P53 signaling pathway were significantly enriched in the MS-high subgroup (**Figures 6A–I**).

### Landscape of genomic variation and expression of different MS subgroups

**Figure 7:**
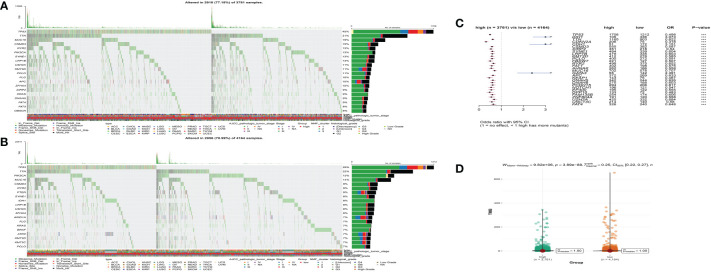
The waterfall plot of tumor somatic mutation of the low- **(A)** and high-MS subgroups **(B)**. **(C)** The differential somatic mutation profile between the low- and high-MS subgroups. **(D)** The TMB status between the low- and high-MS subgroups.

We further analyzed the distribution differences of somatic mutation and TMB between low- and high-MS subgroups in the TCGA cohort. The top 20 genes of mutation frequency of the low- and high-MS subtypes are shown in **Figure 7A, B**, respectively. The top differential mutated genes between the low- and high-MS subtypes are shown **Figure 7C**. Moreover, a remarkable diversity of tumor mutation burden (TMB) was found between the distinct subgroups (**Figure 7D**). The abovementioned results indicated the potentially complex interaction between genomic variation and microbial components in cancers, which might be novel regulators for tumorigenesis and progression.

## Discussion

Increasing evidence suggests microbiome plays an important role in carcinogenesis and progression of various cancer types (24, 39, 40). However, the pan-cancer microbial heterogeneity and their functions are least understood. Here, we conducted a comprehensive analysis of the intratumor microbiota across 32 cancer types, which could provide a better understanding of microbiota dysbiosis and establish a new foundation for studying host-microbiota interactions in tumorigenesis and malignancy in cancer.

We first characterized the global microbial composition at the phylum, class, order, family, and genus levels of tumor and adjacent normal tissues across 32 cancer types. Consistent with previous studies, the relative abundance of Firmicutes and Fusobacteria significantly enriched tumor tissues, whereas that of Proteobacteria, Actinobacteria, Alphaproteobacteria, and Betaproteobacteria were remarkably predominant in adjacent normal tissues (41–47). The LEfSe analysis further supported that microbial taxa, including p_Firmicutes/c_Clostridia, p_Bacteroidetes/c_Bacteroidetes, and p_Fusobacteria/c_Fusobacteria/o_Fusobacteriales, were tumor-enriched microbes, while p_Actinobacteria/c_Actinobacteria, p_Spirochaetes/c_Spirochaetes, and p_Proteobacteria/c_Alphaproteobacteria were normal-enriched microbes, suggesting that distinct microbial component in tumor and normal samples, and specific microbes might play an essential role in tumorigenesis and development of cancer.

Previous studies reported that certain intratumor microbiota of human tumors could also be a potential biomarker for survival outcomes and chemotherapy/immunotherapy (4, 23, 30, 46, 48, 49). Here, we identified specific microbial components that were associated with prognosis in patients from the TCGA cohort. Consistent with other studies, the relative abundance of Actinobacteria, Firmicutes, Fusobacteria, and Proteobacteria were found to be associated with prognosis in cancer. We further established a scoring system (MS) based on candidate microbial signatures that was somehow predictive of survival outcomes, molecular alternation, and immune profiles.

GSVA showed differential genomic function between the high- and low-MS groups. The high-MS group was remarkedly enriched in cell cycle and mismatch repair signaling pathways, while selenoamino acid metabolism and primary bile acid biosynthesis signaling pathways were found significantly enriched in the low-MS group. It is well established that cell cycle and mismatch repair are involved in cancer cell proliferation, sphere-forming capacity, metastasis, and chemotherapeutic/immunotherapeutic sensitiveness (50–54). The interaction between bile acid and microbiota was found to play an essential role in gastrointestinal inflammation and carcinogenesis (55). Considering that, specific microbiomes and their relative biological functions may be involved in the development and malignancy of tumors.

It has been well known that microbiota mediates the host immune system by a complicit mechanism (56, 57). In accordance with previous studies, we demonstrated that microbiota displayed an important role in tumor immune profiles. Furthermore, our study showed that the TMB encounters significant changes between the distinct MS groups. Furthermore, significant gene mutation diversity was observed between the high- and low-MS groups, which indicated the intratumor microbial component might also exert an effect on the immune profile and genomic heterogeneity of tumors.

Compared with recent studies on microbiota alternation in tumors, our research performed a more comprehensive investigation of microbial characteristics in 32 cancer types. However, several limitations need to be clarified in this study. First, the study was analyzed based on the TCGA dataset, so external verification should be performed based on clinical samples to eliminate the false correlation drawing from bioinformatics data in the future. Second, the current study is more of a observational research paper that focuses on the pan-cancer level. The alteration in the microbial community in specific cancer subtypes should be further investigated. Besides, the biological functions and underlying mechanisms of selected microbiota in this study are warranted for further experimental validation.

## Conclusion

In this study, we conducted a comprehensive analysis of the intratumor microbiota in 32 cancer types. Significant differences in the microbial components were found between the tumor and adjacent normal tissues. Several candidate microbial biomarkers were further identified and correlated with tumor prognosis. The potential functions of these microbes in tumors merit further study. Furthermore, we established a microbial-based scoring system, which was significantly related to genomic characteristics, immune features, and survival status of patients in the TCGA cohort. We expect that our research will facilitate a better understanding of the intratumor microbiome and provide a new perspective on the role of the microbiome in tumors.

## Data availability statement

The original contributions presented in the study are included in the article/**Supplementary Material**. Further inquiries can be directed to the corresponding author.

## Ethics statement

The study was approved by the Ethics Institutional Review Board of Sir Run Run Shaw hospital Hospital of Zhejiang University, School of Medicine.

## Author contributions

XY designed, performed the study, and wrote the manuscript. GF, YH, and HA contributed to data and statistical analysis. XY and ZJ supervised the study and revised the manuscript. All authors contributed to the article and approved the submitted version.
